# The midline approach for endotracheal intubation using GlideScope video laryngoscopy could provide better glottis exposure in adults: a randomized controlled trial

**DOI:** 10.1186/s12871-019-0876-6

**Published:** 2019-11-05

**Authors:** Lianxiang Jiang, Shulin Qiu, Peng Zhang, Weidong Yao, Yan Chang, Zeping Dai

**Affiliations:** 1 Department of Anaesthesia, Yijishan Hospital of Wannan Medical College, No. 2, Zheshan West Road, Wuhu City, Anhui Province China; 2 Department of Anaesthesia, Beijing Tiantan Hospital of Capital Medical University, Beijing, China

**Keywords:** Endotracheal intubation, Video laryngosc, Laryngoscopic approach

## Abstract

**Background:**

Previous studies have demonstrated that the common laryngoscopic approach (right-sided) and midline approach are both used for endotracheal intubation by direct laryngoscopy. Although the midline approach is commonly recommended for video laryngoscopy (VL) in the clinic, there is a lack of published evidences to support this practice. This study aimed to evaluate the effects of different video laryngoscopic approaches on intubation.

**Methods:**

Two hundred sixty-two patients aged 18 years who underwent elective surgery under general anaesthesia and required endotracheal intubation were included in the present prospective, randomized, controlled study. The participants were randomly and equally allocated to the right approach (Group R) or midline approach (Group M). All the intubations were conducted by experienced anaesthetists using GlideScope video laryngoscopy. The primary outcomes were Cormack-Lehane laryngoscopic views (CLVs) and first-pass success (FPS) rates. The secondary outcomes were the time to glottis exposure, time to tracheal intubation, haemodynamic responses and other adverse events. Comparative analysis was performed between the groups.

**Results:**

Finally, 262 patients completed the study, and all the tracheas were successfully intubated. No significant differences were observed in the patient characteristics and airway assessments (*P* > 0.05). Compared with Group R, Group M had a better CLV (*χ2* = 14.706, *P* = 0.001) and shorter times to glottis exposure (8.82 ± 2.04 vs 12.38 ± 1.81; *t* = 14.94; *P* < 0.001) and tracheal intubation (37.19 ± 5.01 vs 45.23 ± 4.81; *t* = 13.25; *P* < 0.001), but no difference was found in the FPS rate (70.2% vs 71.8%; *χ2* = 0.074; *P* = 0.446) and intubation procedure time (29.86 ± 2.56 vs 30.46 ± 2.97, *t* = 1.75, *P* = 0.081). Between the groups, the rates of hoarseness or sore throat, minor injury, hypoxemia and changes in SBP and HR showed no significant difference (*P* > 0.05).

**Conclusion:**

Although the FPS rate did not differ based on the laryngoscopic approach, the midline approach could provide better glottis exposure and shorter times to glottis exposure and intubation. The midline approach should be recommended for teaching in VL-assisted endotracheal intubation.

**Trial registration:**

The study was registered on May 18, 2019 in the Chinese Clinical Trial Registry (ChiCTR1900023252).

## Background

In the past decade, video laryngoscopy-assisted tracheal intubation has been extensively applied in airway management because of better visualization of the laryngeal structures on a high-resolution video screen [1–3]. Common teaching in direct laryngoscopy advocates that the device is inserted into the right side of the mouth, the tongue is moved to the left by the blade flange, the blade tip is advanced into the epiglottic vallecula, and then the device is raised to obtain the laryngeal view (right-sided approach) [4]. Until now, this method has been considered the gold standard in tracheal intubation, even for teaching undergraduates. However, we found that the right-sided approach may not be appropriate for intubation using video laryngoscopy (VL) in the clinic. The midline approach without sweep of the tongue is commonly recommended to achieve an unobstructed view of the larynx by VL, but there is a lack of published supporting evidence. Israel and colleagues conducted a retrospective cohort study of children who had undergone endotracheal intubation using VL and found no difference in successful intubation on the first attempt based on the laryngoscopic approach type [5]. However, many factors, including glottis visualization, pre-shaped angulation of the tube, level of trainee and presence of difficult airway predictors, are all correlated with first-pass success (FPS) [6–8]. Other studies have also demonstrated that VL helps to decrease intubation failure but did not improve the FPS in intensive care unit patients requiring intubation or in anaesthesiology practice [9–11]. Therefore, the FPS rate may not be adequate to evaluate the performance of different laryngoscopic approaches for intubation. Urgent evidence is needed to support which approach makes a greater contribution to glottic opening.

In this study, we aimed to compare the right-sided versus midline laryngoscopic blade approach in adult patients who had undergone video laryngoscopy-assisted tracheal intubation using the following outcomes: 1) Cormack-Lehane laryngoscopic views (CLV); 2) first-pass success (FPS) rate; 3) time to glottis exposure and intubation; 4) adverse events and haemodynamic changes during intubation.

## Methods

### Study design

Ethical approval for this study (Ethical Committee NO.4–2019) was provided by the Ethical Issues Committee, Yiji Shan Hospital of Wannan Medical College, Anhui, China (Chairperson Prof Wu P) on March 6, 2019. Written informed consent was obtained from all the patients prior to participation. This study is an interventional, randomized controlled trial and was registered in the Chinese Clinical Trial Registry (ChiCTR1900023252). Our study was adhered to the applicable Consolidated Standards of Reporting Trials (CONSORT) guidelines. The participants were randomly and equally allocated to two groups: right-sided approach group (Group R) and midline approach group (Group M). The randomized sequence was generated by computer, and all allocations were included in sealed opaque envelopes. For randomization, the envelopes will be opened only after transporting the patient to the operating room, and only one envelope can be opened per patient. Because of the nature of the study, the outcome observer could not be blinded to the patients’ group allocation. This was a single-blind clinical trial—that is, patients were blinded to interventions.

Patients older than 18 years, with American Society of Anesthesiology (ASA) physical status I-III, and scheduled to undergo elective surgical procedure under general endotracheal anaesthesia, were all included. Patients were excluded due to the following criteria: 1) patients with a predictable difficult airway: Mallampati score ≥ IV, an interincisor gap less than 3.5 cm, a thyromental distance less than 6.5 cm, a sternomental distance less than 12.5 cm; 2) patients with reduced neck extension and flexion, airway obstruction (infectious, traumatic, foreign body, anaphylaxis), recent airway surgery, or a history of a difficult airway; 3) patients with the need for a rapid sequence induction, an alternative intubation method or known or suspected oral, pharyngeal or laryngeal masses; 4) patients with poor dentition, symptomatic gastro-oesophageal reflux, cervical spine instability, unstable hypertension, coronary artery disease, cerebral disease or patients for whom the resources were not available to conduct the procedure on the scheduled date of surgery.

After transfer to the operative room, the patients were monitored for non-invasive blood pressure (BP), heart rate (HR), pulse oximetry (SpO_2_) and end-tidal carbon dioxide partial pressure (P_ET_CO_2_). The demographic and clinical characteristics of the patients were collected. The patients then underwent a uniform induction technique with midazolam 0.05 mg/kg, propofol 2.0 to 2.5 mg/kg and then were adequately relaxed with cisatracurium 0.15 mg/kg as evident by the loss of all trains of four responses using a peripheral nerve stimulator. With the induction of anaesthesia, the patients could also be administered 0.5 μg/kg of sulfentanyl. All the tracheas were intubated by the oral route using a Glidescope video laryngoscope, size 3 blades (GlideScope® GVL, Verathon Inc., BAothell, WA, USA). For patients in group R, the blade flange was inserted from the right side of the mouth to obtain glottic opening. A midline approach was conducted in group M. In both groups, video laryngoscopy-assisted tracheal intubations were performed by an experienced anaesthesiologist. Intraoperative anaesthesia was intravenously maintained with propofol 4–8 mg/kg/h and remifentanil 0.1–0.2 μg/kg /min. The bispectrality index (BIS) was used to monitor the depth of anaesthesia and keep the BIS value between 45 and 60.

### Outcomes

Our primary outcome was CLV and the FPS rate. The CLV was determined by the modified Cormack-Lehane view of the glottis based on the view obtained at video laryngoscopy: grade I, the glottis is completely visible; grade IIa, the glottis opening is partially visible; grade IIb, only arytenoid cartilage is visible; grade III, only the tip of the epiglottis is visible; and grade IV, no glottis structures are visible [12]. Our secondary outcomes were the times to glottis exposure, intubation procedure time and tracheal intubation time. We defined the time to glottis exposure as the time from the insertion of the blade into the mouth until exposure of the glottis, the time to intubation procedure as the time from finishing exposure of the glottis and ending at blade removal from the mouth and the time to tracheal intubation as the time from starting at blade insertion and ending at blade removal from the mouth. Other outcomes, including hypoxemia (SpO_2_ < 90%), haemodynamic changes [systolic blood pressure (SBP) and heart rate were recorded before intubation, and 1 min, 2 min and 5 min post-intubation], minor injury (oropharyngeal mucosal injury), hoarseness or sore throat on the first postoperative day assessed by a blinded anaesthetist, were also recorded.

### Sample size

We conducted a pilot study of 60 patients for sample size assessment. In this pilot study, the number of CVL grade I-II was 30 (100%) in Group M and 28 (93.3%) in Group R. A sample size of 218 (109 in each group) allowed the detection of a 20% difference between the groups, with an α of 0.05 (two tailed), a β of 0.20, and a power of 0.8. To account for 20% attrition, a total sample size of 262 (131 in each group) was selected.

### Statistical analysis

Continuous variables, such as the height, weight, body mass index (BMI) of the patients and metrics of airway assessments are presented as the mean ± SD. The categorical data are presented as percentages. Ninety-five percent confidence intervals (CIs) for all counts and proportions were also calculated. The primary efficacy variable of the laryngoscopic views, FPS rate and adverse events in different groups were analysed using chi squared test (χ2) or Fisher’s exact test. The Mann-Whitney U test or Student’s t test was used to compare both groups with respect to basic characteristics and other outcomes including the time to glottis exposure, SBP and HR. All the statistical tests were two-sided tests (test level α = 0.05). A *P* value< 0.05 was considered statistically significant.

## Results

**Fig. 1:**
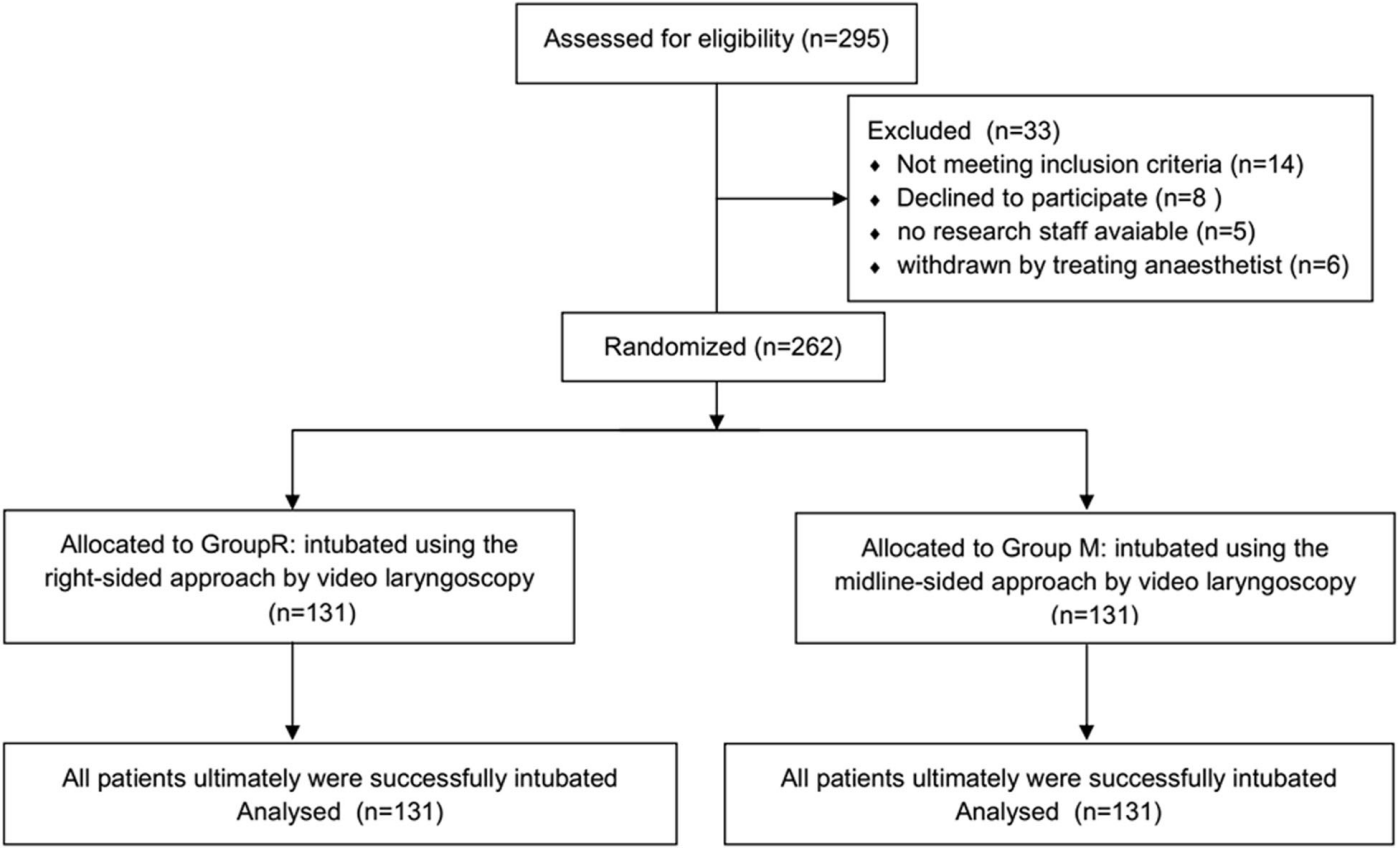
CONSORT flow chart for patient recruitment and randomization

Two hundred ninety-five patients were approached: 14 did not meet the inclusion criteria, 8 declined to participate, and 11 were excluded for other reasons. Finally, 262 patients completed the study, with 131 in each group (Fig. 1). Among them, 133 (50.8%; 95% CI: 44.7 to 56.9%) patients were male and 129 (49.2%; 95% CI: 43.1 to 55.3%) patients were female. The basic characteristics and metrics of airway assessment in both groups are shown in Table 1. No significant differences were observed in the age, gender, weight, height, body mass index, Mallampati score, sternomental distance, interincisor distance, thyromental distance and ASA physical status (*P* > 0.05).

**Table 1:** Patient characteristics and airway assessments

	Group R (*n* = 131)	95%CI	Group M (*n* = 131)	95%CI	p value
Age;year	54.60 ± 11.16	52.67–56.53	56.87 ± 12.60	54.69–59.05	0.124
Gender; %(n)					0.388
Male; %(n)	53.4(70)	44.8–62.1	48.1(63)	39.4–56.8	
Female; %(n)	46.6(61)	37.9–55.2	51.9(68)	43.2–60.6	
Height;cm	165.67 ± 7.11	164.45–166.88	166.27 ± 8.52	164.81–167.73	0.537
Weight;kg	61.46 ± 7.81	60.11–62.81	60.00 ± 8.22	58.58–61.42	0.142
ASA physical status; %(n)					0.455
1	21.4(28)	14.3–28.5	24.4(32)	17.0–31.9	
2	74.0(97)	66.4–81.7	72.5(95)	64.8–80.3	
3	4.6(6)	1.0–8.2	3.1(4)	0.1–6.0	
Body mass index; kg.m^−2^	22.57 ± 3.81	21.91–23.23	21.77 ± 4.01	21.08–22.46	0.119
Sternomental distance;cm	16.52 ± 0.91	16.36–16.68	16.41 ± 1.05	16.23–16.59	0.367
Interincisor distance;cm	3.95 ± 0.56	3.85–4.05	4.05 ± 0.60	3.95–4.15	0.164
Thyromental distance;cm	7.58 ± 0.61	7.48–7.69	7.62 ± 0.58	7.52–7.72	0.587
Mallampati score;%(n)					0.777
1	55.7(73)	47.1–64.3	57.3(75)	48.7–65.8	
2	38.2(50)	29.7–46.6	37.4(49)	29.0–45.8	
3	6.1(8)	2.0–10.3	5.3(7)	1.4–9.2	

Values are Number (proportion) or Mean ± SD. *Group R* right-sided approach group, *Group M* midline approach group, *95% CI* 95% confidence interval

In Group M, 122 (93.1%; 95% CI: 88.7 to 97.5%) patients were grade I, 9 (6.9%; 95% CI: 2.5 to 11.3%) were grade IIa and no patient was above grade IIb. In Group R, 100 (76.3%; 95% CI: 69.0 to 83.7%) patients were grade I, 29 (22.2%; 95% CI: 14.9 to 29.3%) were grade IIa, 2 (1.5%; 95% CI: 0.6 to 3.7%) were grade IIb and no patient was above grade III. Compared with Group R, Group M had a better CLV (*χ2* = 14.706; *P* = 0.001). All the patients’ tracheas were successfully intubated, and the total success rate was comparable in the two groups (*P* = 1.00). Ninety-two (70.2%; 95% CI: 62.3 to 78.2%) patients were successfully intubated on the first attempt in Group M, 94 (71.8%; 95% CI: 63.9 to 79.6%) were successfully intubated on the first attempt in Group R, and the FPS rate showed no difference between the groups (*χ2* = 0.074; *P* = 0.446). Additionally, compared with Group R, Group M had a shorter time to glottis exposure (8.82 ± 2.04 vs 12.38 ± 1.81, *t* = 14.94, *P* < 0.001) and tracheal intubation (37.19 ± 5.01 vs 45.23 ± 4.81, *t* = 13.25, *P* < 0.001), but no difference was found in the intubation procedure time (29.86 ± 2.56 vs 30.46 ± 2.97, *t* = 1.75, *P* = 0.081) (Table 2).

**Table 2:** Details of intubation

	Group R (*n* = 131)	Group M (*n* = 131)	*p* value
Glottic view; %(n)			< 0.001
1	76.3(100)	93.1(122)	
2a	22.2(29)	6.9(9)	
2b	1.5(2)	0	
3	0	0	
FPS rate; %(n)	71.8(94)	70.2(92)	0.446
Total success rate; %(n)	100(131)	100(131)	1.0
Intubation procedure time;s	30.46 ± 2.97	29.86 ± 2.56	0.081
Exposure time;s	12.38 ± 1.81	8.82 ± 2.04	< 0.001
Intubation time;s	45.23 ± 4.81	37.19 ± 5.01	< 0.001
Adverse events; %(n)			
Hypoxemia	4.6(6)	5.3(7)	0.500
Minor injury	6.9(9)	6.1(8)	0.500
Hoarseness or Sore throat	77.9(102)	74.8(98)	0.331

Values are Number (proportion) or Mean ± SD. *Group R* right-sided approach group, *Group M* midline approach group

During intubation, hoarseness or sore throat were the most common adverse events, although the rates of these were not different between the groups (74.8% in Group M vs 77.9% in Group R; *χ2* = 0.338, *P* = 0.331). Seven (5.3%; 95% CI: 1.4 to 9.2%) patients had hypoxemia and 8 (6.1%; 95% CI: 2.0 to 10.3%) had minor injuries in Group M, 6 (4.6%; 95% CI: 1.0 to 8.2%) patients had hypoxemia and 9 (6.9%; 95% CI: 2.5 to 11.3%) had minor injuries in Group R. Additionally, no difference were found in the rates between the groups (hypoxemia: 5.3% vs 4.6%, *χ2* = 0.081, *P* = 0.500; minor injury: 6.1% vs 6.9%, *χ2* = 0.063, *P* = 0.500) (Table 2).

**Fig. 2:**
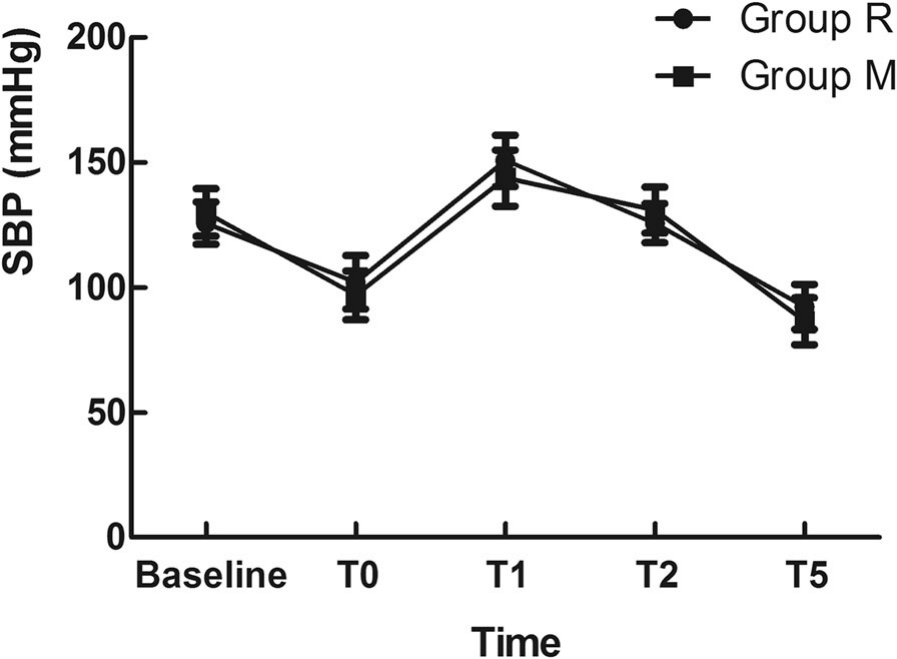
Effect of different laryngoscopic approaches on the systolic blood pressure (SBP)

**Fig. 3:**
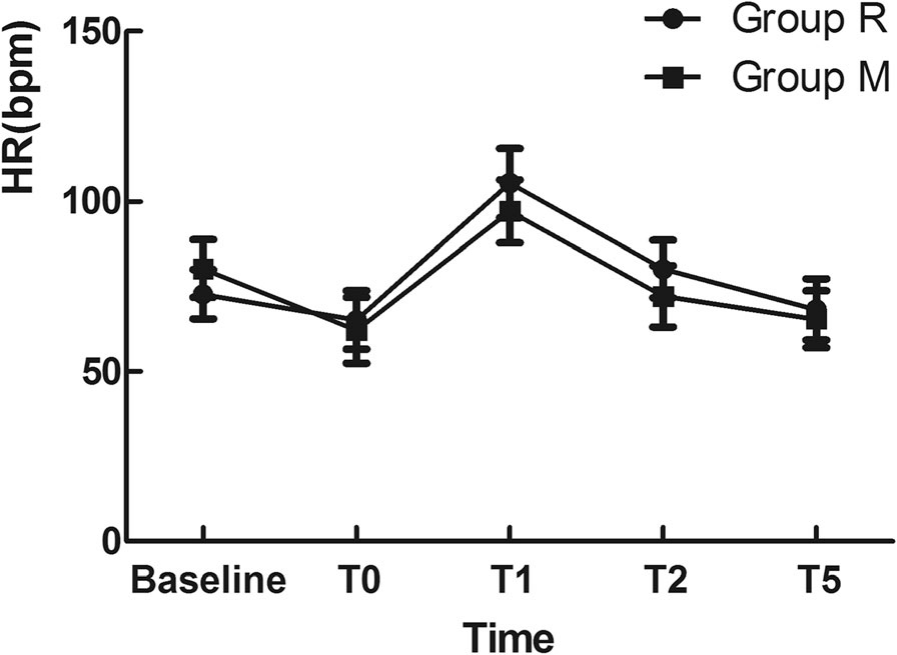
Effect of different laryngoscopic approaches on the heart rate (HR)

Regarding the haemodynamic response to intubation stress, the baseline, SBP and HR before intubation, and at 1 min, 2 min and 5 min post-intubation in the two groups were recorded. No significant difference was found in the changes of SBP and HR between the groups (*P* > 0.05) (Fig. 2 and Fig. 3).

## Discussion

As an available tool for difficult airway management, video laryngoscopy has been demonstrated to improve the success rate and decrease iatrogenic airway trauma [2, 3, 6]. However, the better method of video laryngoscopy-assisted tracheal intubation has not been verified. The performance of intubation using the right-sided approach versus midline approach was compared. We found that the midline approach had better Cormack-Lehane laryngoscopic views and shorter times to glottis exposure and tracheal intubation. The differences in the FPS rate, hypoxemia, haemodynamic response and other adverse events between the groups were not observed.

To obtain adequate direct visualization during intubation, the tongue is commonly swept to the left according to guidance [4, 13]. Although the midline approach has been proposed for intubation by some experts in the early twentieth century, they point to direct laryngoscopy not video laryngoscopy [5, 13–15]. Additionally, no clear evidence exists to support the clinical experience until now. In 2015, Israel and colleagues first explored the effects of both approaches on FPS rate at a paediatric emergency department using the C-MAC video laryngoscope [5]. They found that the PFS rates did not differ based on the laryngoscopic approach type. However, this finding did not illustrate that both approaches were identical because many factors mentioned above contribute to the first-pass success. Similar to their result, the FPS rate was also comparable between the groups in our study. Thus, the success rate of endotracheal intubation was not recognized as our only main outcome. Instead, we believe it was more persuasive to add laryngoscopic views as an indicator to compare the effect of different approaches on intubation. Finally, we demonstrated that glottis exposure in the midline approach was better than the right approach. Additionally, different from both approaches, the left-molar approach requires that the tongue be displaced to the right. It was reported that the left molar approach can also provide a better laryngeal view in cases of unexpected difficult intubation and mitigate difficult intubation when performed by direct laryngoscopy [16, 17]. Nevertheless, whether VL provides a better laryngeal view than the conventional approach requires advanced research.

Because it is difficult to recognize key anatomic landmarks using a right-sided approach, a longer time may be needed to reach an optimal view [5, 6]. Our finding further supports this statement: the midline approach requires a shorter time to glottis exposure than the right-sided approach during tracheal intubation using VL. In the right-sided approach, the tongue was moved to the left and deviated from the median line of the mouth, resulting in angulation between the tongue and blade. Much more force was needed to expose the glottis due to the dispersion of forces. By contrast, in the midline approach, the laryngoscope blade is straight to expose the glottis, avoiding more forces. Adverse events such as oedema, tooth trauma, and soft tissue lesions can be caused by excessive forces transmitted through the laryngoscope during an intubation [18]. Thus, the rate of minor injury in the midline approach was lower than that in the right approach in theory. Increasing evidence has indicated that the video laryngoscope has an established role in tracheal intubation, decreasing the forces applied to the soft tissues of the upper airway and incidence of complications, compared with the Macintosh laryngoscope [18–20]. However, we found no difference in the rates of adverse events between the approaches in contrast to these studies and Israel’s results. Two explanations are possible. First, patients with predictable difficult airways were not included in our study. All the endotracheal intubations in both groups were conducted by VL with a lower applied force. Second, the participants were children in Israel’s study while they were adults in the present study. Compared with the adults, the children may be more likely to suffer from tissue damage. For the haemodynamic response to intubation, the patients did not significantly differ in SBP and HR after stress between the groups.

Our study possessed limitations. First, video laryngoscopy was originally designed as a device to manage difficult intubation with direct laryngoscopy, but the patients with predicable difficult airways were all excluded in our trial. Whether the midline approach would be effective in this population needs further study. Second, investigators were not blinded to the outcome measures. Third, several video laryngoscopes with different designs are commercially available and have been investigated in various settings [21, 22]. All the intubation procedures were performed using a Glidescope video laryngoscope in the present study. Whether the results were applied to other video laryngoscopes may be worth discussing.

## Conclusions

We observed that the midline approach was associated with a better glottis exposure and a shorter time to intubation than the right-sided approach. The midline approach should be recommended for teaching in video laryngoscopy-assisted endotracheal intubation.

## Data Availability

The datasets used and analysed during the current study are available from the corresponding author on reasonable request.

## Abbreviations

ASA: American Society of Anesthesiology
BIS: bispectral index
BMI: Body mass index
BP: Non-invasive blood pressure
CLV: Cormack-Lehane laryngoscopic views
FPS: First-pass success
HR: Heart rate
P_ET_CO_2_: End-tidal carbon dioxide partial pressure
SBP: Systolic blood pressure
SpO_2_: Pulse oximetry
VL: Video laryngoscopy
